# Breaking the Blister: A Case Report of Bullous Wells’ Syndrome Resolved with Oral Terbinafine

**DOI:** 10.7759/cureus.80060

**Published:** 2025-03-04

**Authors:** Winnie W Bao, Eunice Y Chow

**Affiliations:** 1 Medicine, University of Alberta, Edmonton, CAN; 2 Medicine/Dermatology, University of Alberta, Edmonton, CAN

**Keywords:** blood eosinophils, cellulitis, dermatitis, eosinophilic cellulitis (ec), flame figures, rare skin disease, wells’ syndrome

## Abstract

Eosinophilic cellulitis (Wells' syndrome) is a rare inflammatory dermatosis, characterized by erythematous skin lesions, edema, and eosinophilic infiltration into the dermis. While typically affecting the limbs, it is uncommon for the condition to present as bullae on the feet. Most cases are idiopathic; however, triggering factors may include arthropod bites, certain medications, and viral, bacterial, or parasitic infections. In this case, a 52-year-old woman presented with recurrent pruritic, blistering eruptions on her feet over the past year. The lesions were non-responsive to multiple treatments, including topical and oral steroids, several antibiotics, and methotrexate. She was diagnosed with eosinophilic cellulitis by histopathology, possibly triggered by tinea pedis and onychomycosis. A three-month course of oral terbinafine resolved her fungal infection and recurrent eosinophilic cellulitis. This atypical presentation, including persistent scaly patches and bullae resistant to known treatments for eosinophilic cellulitis, highlights the challenges clinicians face when diagnosing this condition among a broad differential. It should be considered for any unexplained recurrent cellulitis-appearing inflammatory skin condition, not responsive to usual therapy. Additional triggers of eosinophilic cellulitis should be considered beyond those already identified in the literature, such as tinea pedis and onychomycosis as presented in this unique case. Addressing the underlying cause may lead to complete remission of eosinophilic cellulitis.

## Introduction

Eosinophilic cellulitis (EC), also known as Wells’ syndrome, is a rare dermatological condition, first described in 1971 [1]. To date, a few hundred cases have been documented in the literature [2]. This condition is characterized by recurrent erythematous skin lesions typically on the extremities and/or trunk. In the acute phase, these lesions may present as pruritic, burning, tender, or edematous, resembling cellulitis, but are non-responsive to antibiotics. Hallmark histological features include eosinophilic infiltration into the dermis, dermal edema, absence of vasculitis, and “flame figures”, which are areas of degranulated eosinophils coating collagen bundles [2]. During the resolving phase, lesions progressively improve over two to eight weeks, but hyperpigmentation and scarring may persist [3].

Although its etiology remains elusive, EC has been associated with various triggers, including arthropod bites, medications, malignancies, viral and parasitic infections, and autoimmune disorders [4-6]. In this report, we describe the case of a 52-year-old woman with an atypical presentation of EC: recurrent bullae of the feet, precipitated by tinea pedis and suspected onychomycosis, successfully treated with oral terbinafine.

## Case presentation

A 52-year-old female patient presented to the dermatology clinic seeking a second opinion in regard to a one-year history of recurrent pruritic and tender, blistering eruptions, primarily around the dorsal and plantar surfaces of her second to fifth toes. These eruptions first appeared as pruritic macules and papules but would progress to large, tender, and pruritic blisters that would affect one or both of her feet at a time. Episodic flares seemed to occur monthly on alternating feet. No ocular symptoms, additional lesions, or sun-sensitive rashes were reported. She denied any new exposures to her feet, such as new footwear or topical agents. However, she did start going to the swimming pool regularly shortly before the onset of the eruption. The blisters were not affected by temperature, and she continued to develop the blisters year-round, even while visiting warm locales. 

Her past medical history included epilepsy and osteoarthrosis. She took a daily multivitamin and used a levonorgestrel intrauterine device (IUD). Allergies to topical dexamethasone eye drops and moxifloxacin were noted. She was originally given the diagnosis of dyshidrotic eczema, then cellulitis by her first doctor after her right foot became warm, erythematous, and swollen transiently. However, the lesions were refractory to multiple treatments with adequate trials, including topical steroids (betamethasone valerate 0.1% cream and clobetasol 0.05% ointment), terbinafine 1% spray for one week, azithromycin 250 mg once daily for three months, cephalexin 500 mg four times daily for 10 days, and clindamycin 150 mg twice daily for 14 days. Finally, she was prescribed methotrexate 15 mg/week but she continued to develop recurrent bullae to the feet. Thus, she was given a concurrent five-day course of prednisone 50 mg/day, which seemed to help, only to have the bullae recur after the prednisone course was finished. Despite an increase of methotrexate to 25 mg/week, the lesions persisted, and so she was prescribed additional prednisone 15 mg/day, only to stop it after a few days due to gastrointestinal side effects. The total duration of methotrexate therapy was 19 weeks. The only alleviating factor was soaking her feet in hot spring water.

**Figure 1:**
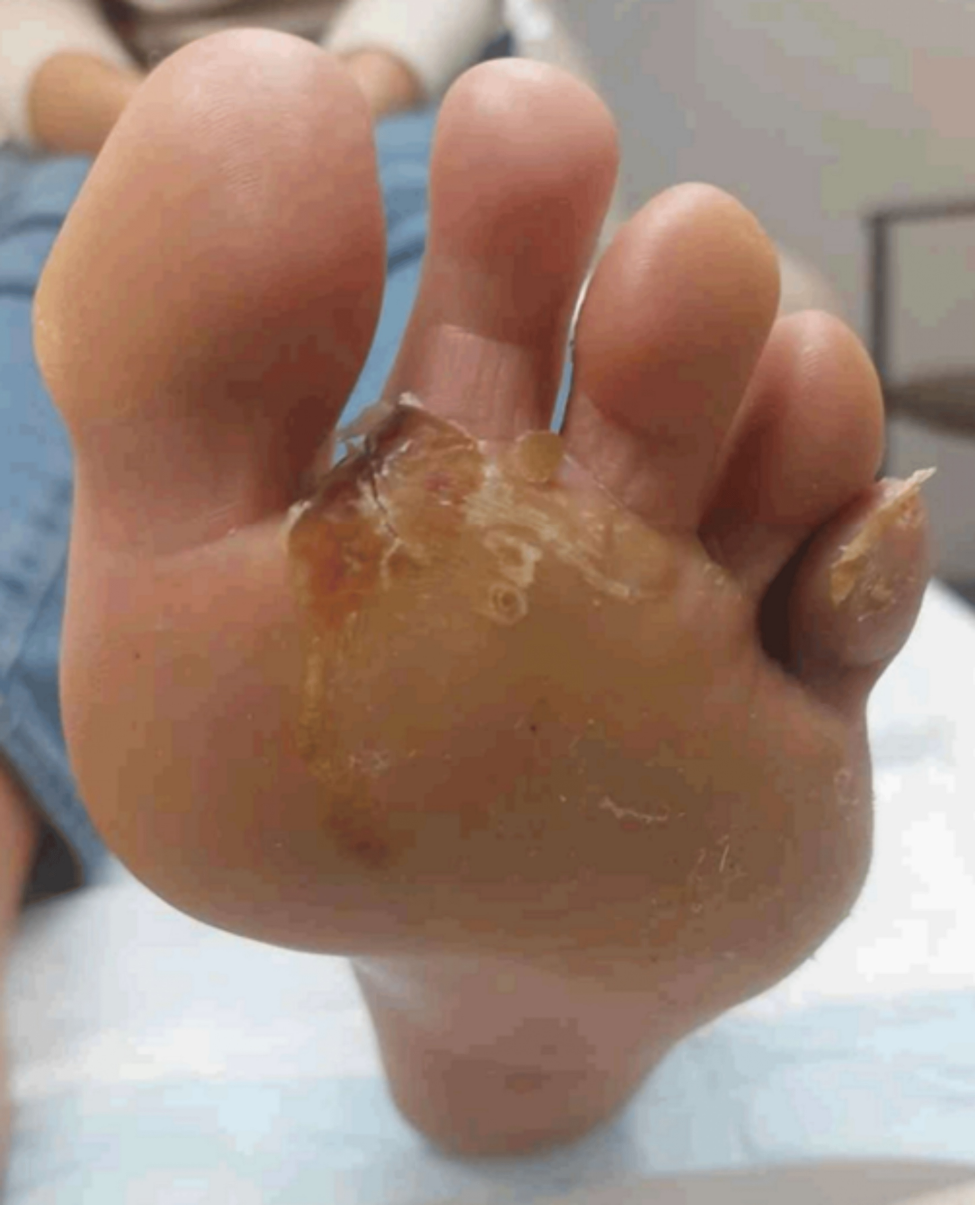
Recently resolved, healing bulla on the left plantar foot at the initial dermatology consultation.

**Figure 2:**
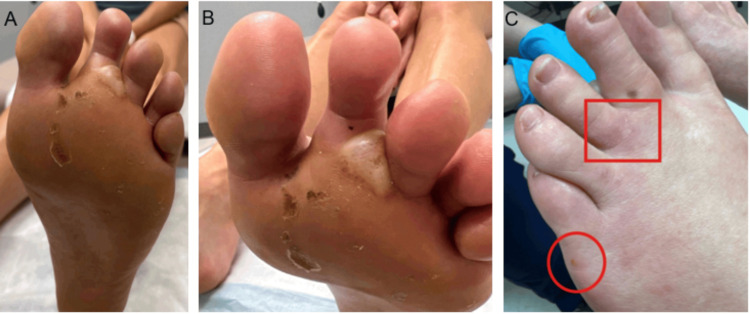
Recurrence of the lesions on the plantar (A-B) and dorsal (C) surfaces of the left foot at a follow-up visit, one month after the initial consult. (A-B) Large subepidermal bulla at the base of the D2 and D3 toes, with scales in the forefoot and within the flexor crease of the fourth toe. (C) Pruritic and tender ill-defined erythematous indurated patches and plaques, with a small 1-2mm yellow vesicle on the lateral aspect. Punch biopsies were taken at the level of the MTP (red circle) and at the base of the D3 toe (red square). MTP: metatarsophalangeal

At the initial dermatology visit, there was evidence of a healing bulla with a dried yellowed crusted patch at the base of the second left toe (Figure 1). Investigations were not initiated at this time since the lesion was in a state of resolution. However, one month later, she presented with an active episode of a large 1.2 cm tense bulla filled with clear, yellow fluid on the plantar aspect of her left foot at the base of the second toe (D2) (Figures 2A-2B). At the base of the dorsal left third toe (D3), there was an ill-defined erythematous, mildly indurated 1.5 cm plaque. On the lateral side of the foot, at the level of the metatarsophalangeal (MTP) joint, a minute 1-2 mm yellow vesicle was observed on a background of ill-defined erythema (Figure 2C). The differential diagnoses included bullous tinea, bullous impetigo, EC, acute febrile neutrophilic dermatosis, autoimmune connective tissue diseases, epidermolysis bullosa acquisita, pemphigus vulgaris, dyshidrotic eczema, and pompholyx. Three 3 mm punch biopsies were taken from these sites and sent for histopathology. An additional punch biopsy adjacent to the bulla on the left plantar foot was collected and sent for direct immunofluorescence.

**Figure 3:**
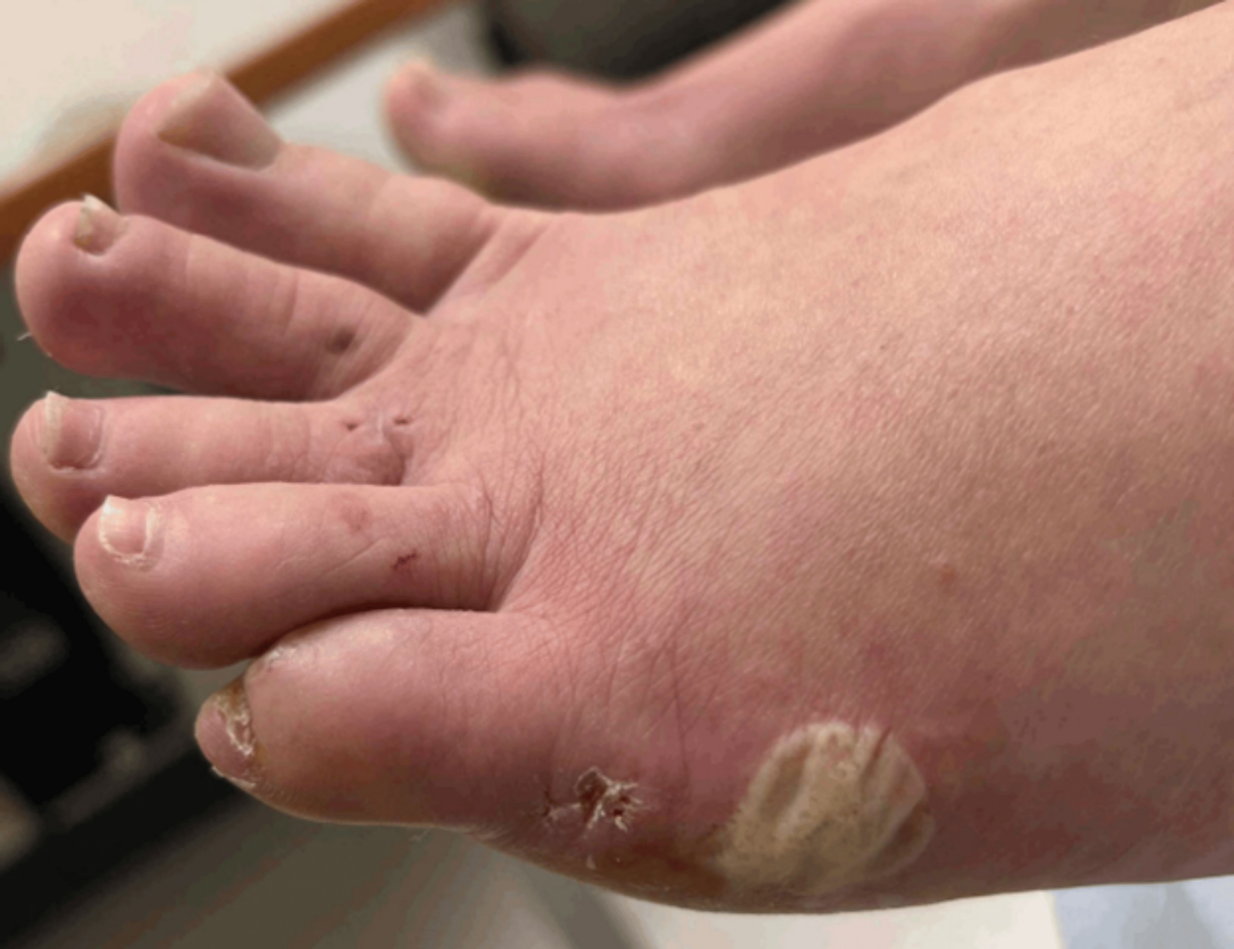
Resolving bulla over the left lateral foot inferior to a previous biopsy site, two weeks after the punch biopsies.

Bacterial and viral swabs (herpes simplex virus and varicella-zoster virus) from the punctured bulla were negative for infection. Laboratory investigations demonstrated a minimally elevated eosinophil count (1.1 x 10^9^/L). Previous prick testing indicated an allergy to bees, wasps, and hornets. To rule out allergic contact dermatitis, patch testing was conducted with the North American 80 Comprehensive Series (Dormer Laboratories Inc., Toronto, Canada) in addition to the shoe series, fragrance series, cosmetic series, and a University of Alberta liquid series. A total of 205 haptens were tested and resulted in positive responses to benzoic acid and butylhydroquinone, but were deemed irrelevant to her foot eruption. A skin scraping was taken for fungal culture and exam from the left plantar foot which yielded a positive culture for *Trichophyton rubrum*. Sensitivity testing for dermatophytes is not routinely reported by the laboratories in the health zone. While awaiting the biopsy results, the patient began a course of oral terbinafine 250 mg once daily for two weeks to treat her tinea pedis. Within two days, she started to notice improvement of the lesions on the right foot, but continued to develop a bulla on the left foot (Figure 3).

**Figure 4:**
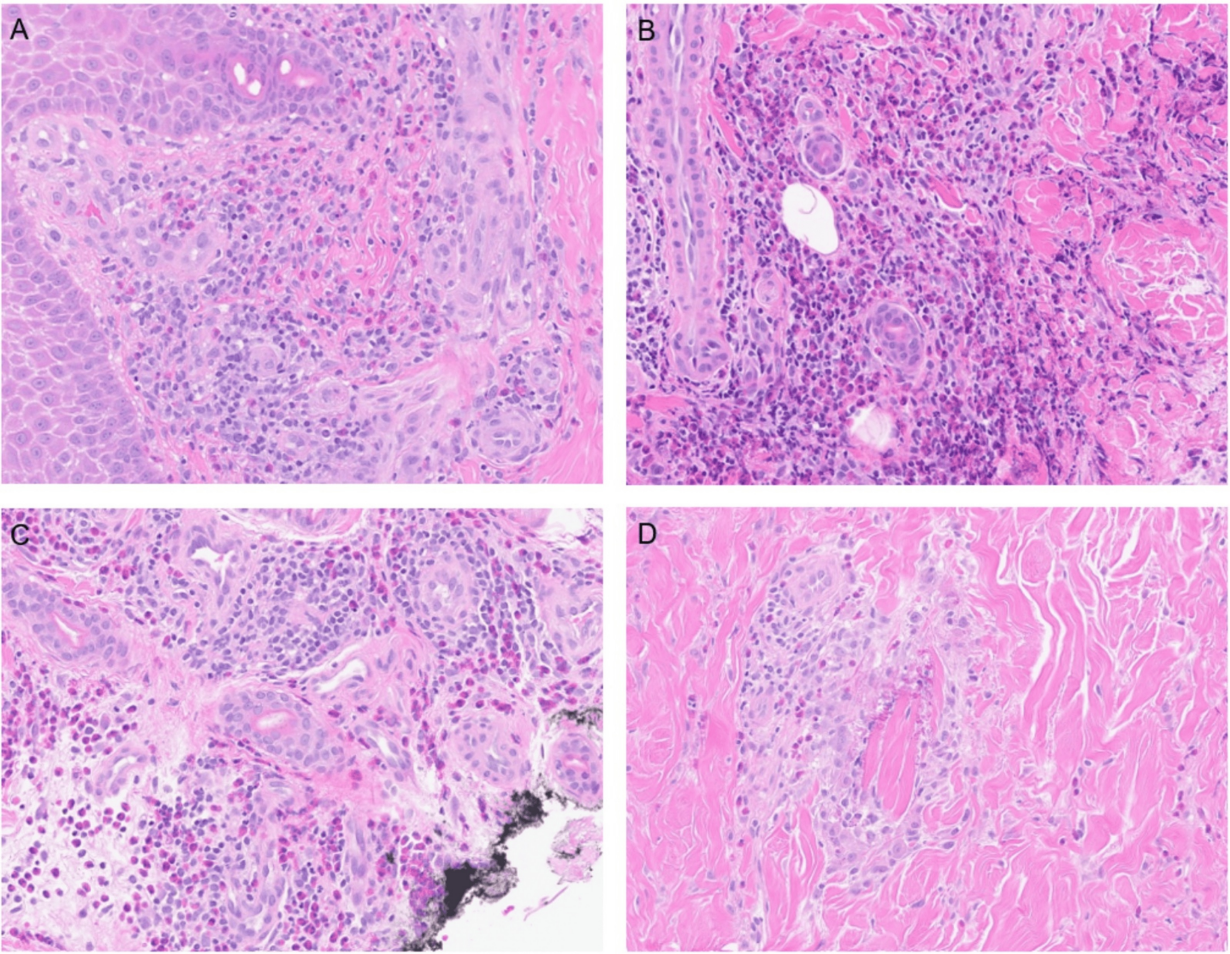
Histopathology H&E 400x Pathological results demonstrate prominent eosinophils in the dermis (A-B) and flame figures (C-D), consistent with eosinophilic cellulitis.

All three skin specimens sent for histopathology showed intense dermal eosinophilic infiltrate with flame figures, suspicious for EC (Figure 4). The direct immunofluorescence was negative, and the Periodic Acid-Schiff plus Diastase (PAS-D) stains were negative for fungi. The patient finished the two-week course of terbinafine and started ciclopirox cream, bilastine 40 mg twice daily, and prednisone 30 mg once daily for five days. The lesions resolved more quickly, and the blisters did not recur as frequently after the discontinuation of prednisone. Despite completing treatment, the patient still experienced recurrence of a few small bullae on her feet.

**Figure 5:**
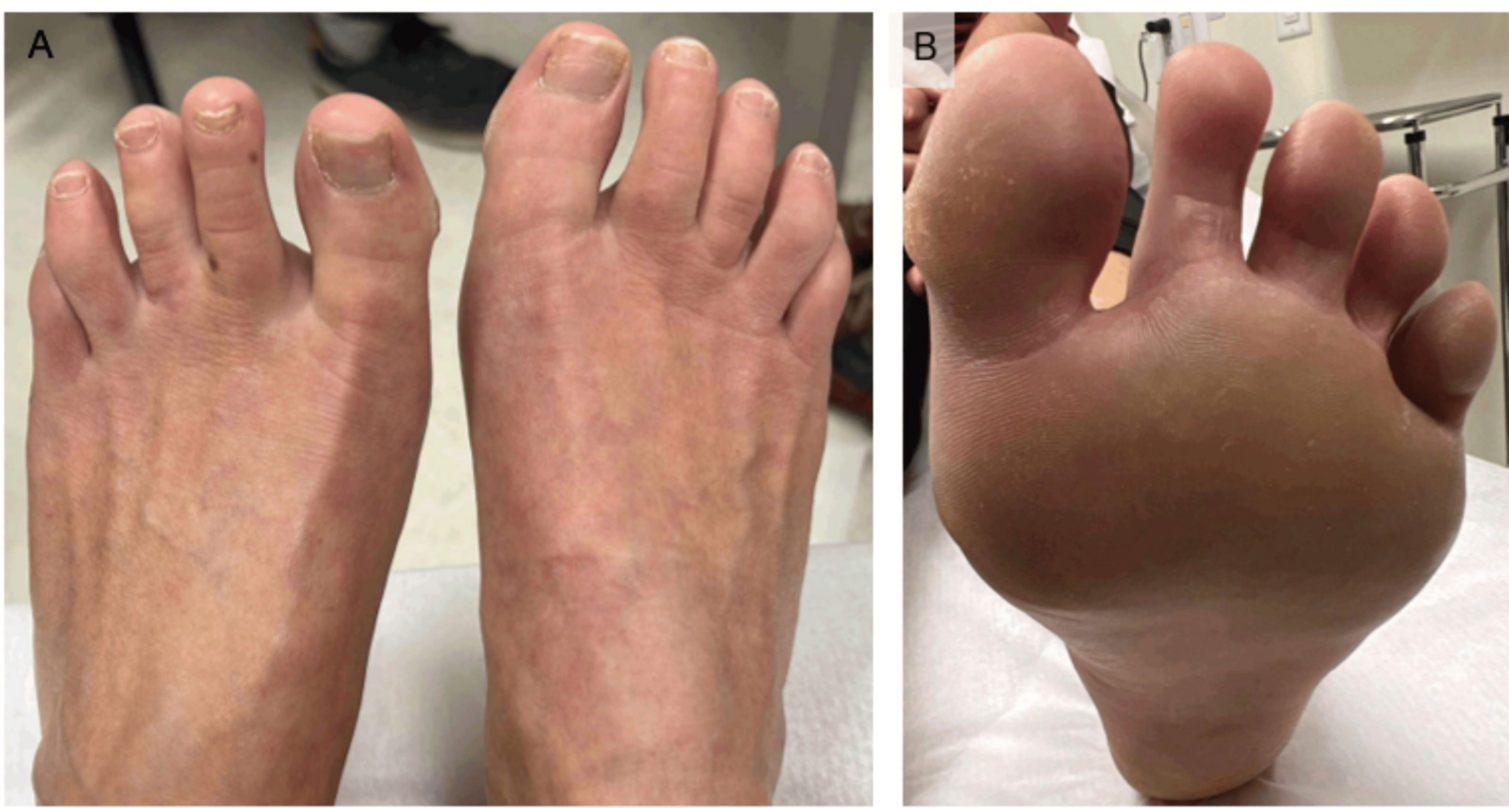
Resolution of the lesions on the dorsal (A) and plantar (B) feet, one month after the completion of the three month course of oral terbinafine. (A) Clear skin on the dorsum of the feet bilaterally. (B) Resolution of the bulla and scaling of the plantar surface of the left foot.

**Figure 6:**
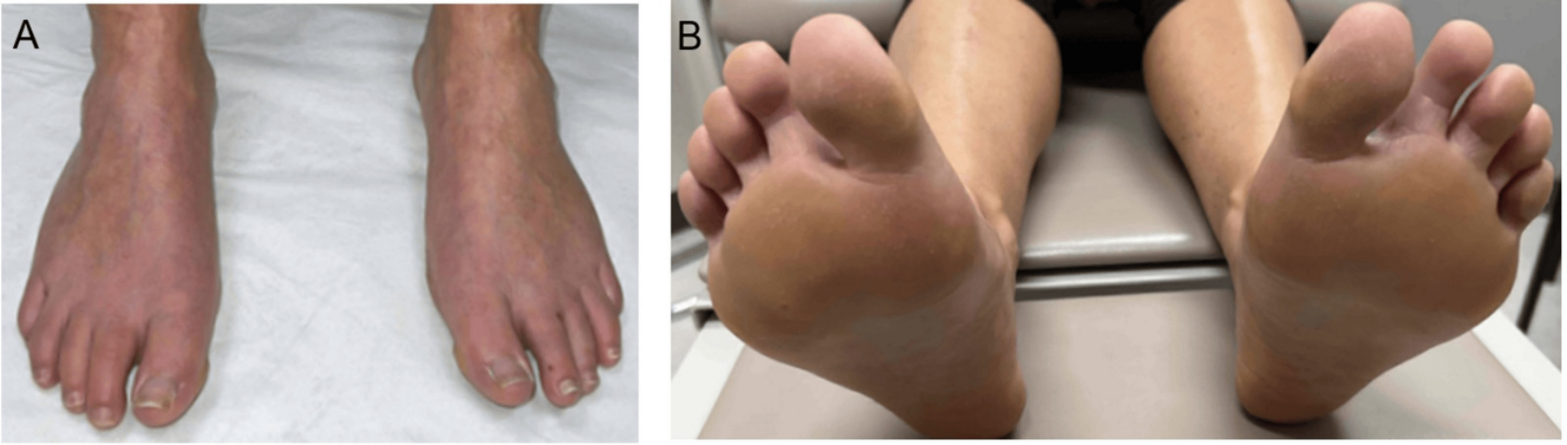
Bilateral dorsal (A) and plantar (B) feet showing no recurrence of eosinophilic cellulitis, approximately one year after the completion of the three month course of oral terbinafine.

On further examination, the patient had yellow discolored toenails on the great toes (D1) bilaterally with distal onycholysis in addition to onychauxis and yellow discoloration of the left second (D2) and fifth (D5) toenails. Nail clippings of the affected toenails failed to grow dermatophytes. However, based on the positive skin scraping culture of the feet, the appearance of the nails, and the fact that she had already taken two weeks of oral terbinafine with good effect, it was decided that the patient would complete a three-month course of oral terbinafine 250 mg/day for presumed onychomycosis. No other treatments were used concurrently during this time. Within the first month of the oral terbinafine course, there was complete resolution of the bulla with no recurrence (Figure 5). Over a period of one and a half years, the patient has not reported any further episodes of EC to date (Figure 6).

## Discussion

EC, a rare inflammatory dermatosis, is characterized by erythema, edema, and cutaneous lesions with eosinophilic infiltration into the dermis. Although the condition typically affects the extremities, it can also involve other regions of the body, including the trunk and face [2]. This case is particularly noteworthy due to the localization of EC on the feet. In addition, the presence of bullae in the context of EC further distinguishes this case. Bullous EC, while recognized in the literature, is a rare manifestation of the disease [7]. This uncommon presentation may pose a diagnostic challenge, necessitating a comprehensive evaluation to rule out other conditions that may appear similarly on the feet such as bullous cellulitis, blistering pernio/chilblains, dyshidrotic eczema, bullous tinea pedis, bullous impetigo, bullous allergic/irritant contact dermatitis, localized bullous pemphigoid, dyshidrosiform bullous pemphigoid, epidermolysis bullosa acquista, or porphyria/pseudoporphyria.

Other EC lesion variants include plaque-type, annular granuloma-like, urticaria-like, papulovesicular, papulonodular, and fixed drug eruption-like [6]. This diversity in presentation supports EC as an important differential diagnosis for any related cutaneous eruption associated with eosinophilic infiltration of the dermis.

Histopathological findings remain essential in diagnosing EC, as its clinical features can overlap with many other inflammatory or infectious skin diseases. In this case, a skin biopsy revealed the characteristic eosinophilic dermal infiltration and flame figures, confirming the diagnosis. Along with histopathology, peripheral eosinophilia and elevated levels of eosinophilic cationic protein may be seen, although these markers are not always definitive [8]. Given the rarity and nonspecific nature of the disease, a skin biopsy should be performed in ambiguous cases to ensure an accurate diagnosis and to rule out other differentials. 

In 2013, Heelan et al. proposed diagnostic criteria for EC which required two of the four major criteria: (i) diverse clinical picture to include any of the previously reported variants (plaque-type, annular-granuloma-like, urticaria-like, papulovesicular, bullous, papulonodular and fixed drug eruption-like), (ii) relapsing, remitting course, (iii) no evidence of systemic disease, and (iv) eosinophilic infiltrates and no vasculitis on histology, plus at least one minor criterion from: (i) flame figures, (ii) granulomatous change on histology, (iii) peripheral eosinophilia not persistent and not greater than >1500/μl, and (iv) triggering factor [6].

The pathophysiology of EC is unclear, but it is hypothesized to involve a type IV hypersensitivity reaction mediated by eosinophils [9]. Cytokines such as interleukin-5 are thought to play a central role in eosinophil activation, tissue infiltration, and degranulation [8]. Most EC cases are idiopathic; however, various precipitating factors have been reported, including medications, bacterial, viral, or parasitic infections, hematological malignancies, arthropod bites and stings, metals, and vaccinations [10]. 

A unique aspect of this case is the identification and management of a potential trigger for EC, ultimately leading to its remission. It was suspected that the patient developed tinea pedis and onychomycosis after visiting the pool more regularly, which precipitated her EC. Treatment of her tinea pedis, and likely onychomycosis, with oral terbinafine, resulted in the resolution of her recurrent EC. Although we were not able to culture the *Trichophyton rubrum* in the nail plate clippings, it may have been partially due to the fact that she had already taken two weeks of oral terbinafine in addition to ongoing use of topical antifungal creams prior to the nail culture. Tinea pedis is a known risk factor for onychomycosis and *Trichophyton rubrum* is the most common cause of dermatophyte onychomycosis [11]. Finally, the reported sensitivity of nail culture is only 31-59% [11], leaving a high rate of false-negative culture results. In this case, identifying the potential infectious trigger was paramount to the targeted therapy with oral antifungals that led to the resolution of her recurrent episodes of EC, and may explain why immunosuppressive therapies alone such as methotrexate and prednisone were ineffective in the resolution of her EC. 

Considering the uncommon occurrence of EC, treatment recommendations are limited to case reports and series. A variety of options have been documented such as topical and systemic corticosteroids, antihistamines, antibiotics, antimalarial medications, cyclosporine, azathioprine, colchicine, and methotrexate [12]. In a series of 32 EC cases, oral steroids were reported as the most effective therapy, with a resolution rate of 92% [2]. Topical corticosteroids and antihistamines had lower success rates of 50% and 25%, respectively. Spontaneous resolution of lesions was seen in 12.5% of cases. For the patient in the current case, these treatment modalities failed to provide complete clearance. At a higher dose of prednisone (50 mg/day), the lesions resolved, only to return shortly after the discontinuation of prednisone. Other treatment options, such as topical corticosteroids and antihistamines, also did not improve the lesions.

Unfortunately, EC often follows a recurrent pattern. Sinno et al. reported its recurrence rate as 56% in a case series [2]. On average, episodes reoccurred every six months; however, a wide range existed from three weeks to 24 months [2]. Another distinctive aspect of this case is the high frequency of flares on a monthly basis for over a year. Only one case in the case series described recurrent episodes to this degree [2]. The recommended treatment for recurrent EC includes low-dose prednisone and steroid-sparing agents, such as methotrexate [13]. Our patient could not tolerate low-dose prednisone and methotrexate was ineffective in resolving her EC.

Despite its benign course, EC may significantly impact a patient’s quality of life due to its recurrent nature and potential for misdiagnosis. Further research is necessary to elucidate the underlying etiology and develop therapeutic guidelines.

## Conclusions

This case report contributes to the growing body of literature on EC, a rare and often misdiagnosed condition. Misdiagnosis leads to treatment delays, adverse patient outcomes, and unnecessary burden on the healthcare system. The unique features of this case, such as plantar bullae refractory to usual therapy and the identification of a trigger, emphasize the challenges clinicians face when diagnosing EC among a broad differential. EC should be considered for any unexplained recurrent cellulitis-appearing inflammatory skin condition, not responsive to usual treatment. A comprehensive diagnostic approach is essential and includes careful consideration of clinical presentation, histopathologic findings, and potential underlying causes.
